# miR-543 impairs breast cancer cell phenotypes by targeting and suppressing ubiquitin-conjugating enzyme E2T (UBE2T)

**DOI:** 10.1080/21655979.2021.2005217

**Published:** 2021-12-11

**Authors:** Li Li, Qing Li

**Affiliations:** a Department of Thyroid and Breast Surgery, The Affiliated Hospital of Jianghan University, Wuhan Hubei, China; b Department of Oncology, The Affiliated Hospital of Jianghan University, Wuhan Hubei, China

**Keywords:** MIR-543, UBE2T, breast cancer, ERK/MAPK pathway, MCF-7, MDA-MB-231

## Abstract

Breast cancer, with high morbidity worldwide, is a threat to the life of women. MiR-543 was identified as playing an active part in the development of breast cancer involving multiple molecules. The goal of this study was to explore the molecular mechanisms of the involvement of miR-543 in the development of breast cancer. Quantitative real-time PCR (qRT-PCR) or Western blotting was used to detect mRNA or protein expression. Cell counting kit-8 (CCK-8), and the 5-bromo-2ʹ-deoxyuridine (BrdU), wound healing, and Transwell assays were the main experimental procedures. Furthermore, subcutaneous tumor formation experiments were conducted to detect the function of miR-543 in breast cancer development in vivo. The match of miR-543 and ubiquitin-conjugating enzyme E2T (UBE2T) was detected through a dual-luciferase reporter experiment and RNA pull-down assay. Based on these results, miR-543 exhibited reduced expression in breast cancer tissues and cell lines, whereas UBE2T exhibited high levels. Furthermore, miR-543 directly targeted UBE2T, and a negative correlation between miR-543 and UBE2T was also observed in breast cancer tissues. Moreover, miR-543 overexpression led to inhibition of viability, proliferation, migration, and invasion of breast cancer. Furthermore, miR-543 overexpression undermined the UBE2T promotional effect by inhibiting ERK/MAPK pathway activity in breast cancer cells. Our study revealed that miR-543 impaired breast cancer progression by targeting UBE2T and downregulating UBE2T expression through the ERK/MAPK pathway, which suggested that miR-543 and UBE2T might serve as promising therapeutic gene targets for breast cancer in clinical application.

## Introduction

Despite pivotal advances in cancer treatment, breast cancer remains a major health problem worldwide and has become a priority in biomedical research [1,2]. This aggressive disease is threatening the lives of thousands of women with alarmingly high incidence rates that suggest more progress should be made in its prevention and diagnosis [3,4]. Over the years, miRNA is attracting the attention of researchers in the search for cancer treatment strategies. Here, we attempted to determine the mechanism by which this non-coding RNA affects breast cancer to discover a therapy for breast cancer.

Over the past decade, miR-543 has been demonstrated to be a novel miRNA highly related to breast cancer pathological progress. A growing body of literature recognizes miR-543 as a promising potential prognostic and diagnostic biomarker in clinical therapy. In 2012, miR-543 was identified as having lower expression in breast cancer in a microarray analysis [5]. In 2017, Chen et al. found that miR-543 inhibited breast cancer progression via direct targeting of ERK2 in the MARK/ERK signaling pathway, which provided a molecular basis for improved treatment for patients with breast cancer [6]. More recently, Li and colleagues illustrated that miR-543 through suppression of VCAN impaired breast cancer cell proliferation, migration, and invasion [7]. Based on the results for miR-543 as a clinical biomarker in breast cancer, the additional molecular mechanisms underlying miR-543 in the regulation of breast cancer progression should be explored.

Ubiquitin-conjugating enzyme E2T (UBE2T) belongs to the ubiquitin-proteasome family, which is in charge of various cell behaviors, such as DNA replication, cell growth, and angiogenesis [8–10]. Initially, UBE2T was identified in a case of Fanconi anemia [11]. Later, the cancer-inducing role of UBE2T was demonstrated in multiple types of malignancies, including breast cancer. For example, higher UBE2T expression levels were predictive of a lower pathological complete response rate in patients with triple-negative breast cancer [12]. Additionally, it was shown that UBE2T played a critical role in the development and/or progression of breast cancer through the interaction with and the regulation of the BRCA1/BARD1 complex [13]. Furthermore, another study suggested that UBE2T had a role in the pathophysiology of breast and lung tumors and affirmed the function of UBE2T in future clinical treatments for patients with breast cancer [14]. Liu et al. found that the miR-543/UBE2T/p53 axis represents a potential new therapeutic target for hepatocellular carcinoma intervention [15], which provides evidence miR-543 directly targets UBE2T. However, the mechanism of UBE2T and miR-543 in regulating breast cancer development and progression has not been explored. Interestingly, it has been reported that miR-543 is related to the MAPK signaling pathway in breast cancer [5]. The MAPK/ERK pathway is a significant signaling cascade among all MAPK signal transduction pathways and plays an important role in the survival and development of tumors (reviewed by [16]). Additionally, Chen et al. reported that miR-543 suppressed breast cancer malignancy by inhibiting the ERK/MAPK pathway [6]. However, no study has addressed the effects of UBE2T on the ERK/MAPK pathway.

Here, the purpose of this study was to explore the relationship between UBE2T and miR-543 and their regulation pathway in breast cancer progression. Our results suggested that the miR-543/UBE2T axis and ERK/MAPK pathway be crucial in breast cancer diagnosis and provide new breast cancer treatment insights.

## Materials and methods

### Bioinformatics analysis

GSE113740 and GSE146477 were downloaded from the GEO datasets, including the miRNA expression profile in breast cancer and non-tumor samples. With an adjusted P < 0.05, |log_2_FC|≥1, the differently expressed miRNAs in breast cancer samples were screened out. GEPIA is a database including the mRNA expression profile in breast cancer samples and non-tumor samples. The upregulated genes in breast cancer samples were screened out With an adjusted P < 0.05, log_2_FC≥1.5. Additionally, the targetscan algorithm (http://targetscan.org/vert_72) was used to predict the target genes of key miRNA, and the STRING database was used to analyze the protein-protein interaction (PPI) network for the identification of the key genes.

### Tissues

With written informed consent, the paired breast cancer tissues and adjacent healthy tissues were collected from 36 patients with breast cancer in our hospital from 2019 to 2020. The study was approved by the Ethics Committee of our hospital (approval number: WHSHIRB-K-2020023) and was conducted following the World Medical Association Declaration of Helsinki.

### Cell culture

Human breast cancer lines (HCC1937, MCF-7, MDA-MB-231) and human normal mammary epithelial cells (MCF-10A) were purchased from the Cell Resource Center, Institute of Basic Medical Sciences, Chinese Academy of Medical Sciences (Beijing, China) and cultured as previously described [17].

### Cell transfection

Before transfection, 2.5 × 10^6^ or 1 × 10^5^ breast cancer cells were incubated using 6‐well or 96-well plates with 2 mL or 10 μL complete medium for 24 h until they were 80% confluent. miR-543 mimics, UBE2T overexpression plasmids, and negative controls (NCs) were purchased from GenePharma (Shanghai, China). As reported previously [6], the Lipofectamine 2000 reagent (Invitrogen, USA) was used for the transfection.

### qRT-PCR

MagZol Reagent (Magen, Guangdong, China) was used to extract total RNA. The miRNA 1st Strand cDNA Synthesis Kit (by stem-loop) (Vazyme, China) was used for reverse transcription PCR for miRNA. mRNA reverse transcription was conducted using the BeyoRT™ II First Strand cDNA Synthesis Kit with gDNA Eraser (Beyotime, China). The quantification levels of miR-543 and UBE2T were conducted with GAPDH and U6 as internal controls, respectively, using the 2^−ΔΔCt^ method [18]. The primers are depicted in Table 1.

**Table 1.** The primers for RT-qPCR for each gene

Gene name	Primer sequence	
UBE2T	forward	5ʹ-CGAGCTCGTAGAAATATTAGGTGGA-3’
	reverse	5ʹ-TCATCAGGGTTGGGTTCTGAC-3’
miR-543	forward	5` – CAGTGCTAAAACATTCGCGG – 3`
	reverse	5ʹ-TATGGTTGTTCACGACTCCTTCAC-3’
GAPDH	forward	5′-GAGAAGGCTGGGGCTCATTT-3′
	reverse	5′-AGTGATGGCATGGACTGTGG-3′
U6	forward	5′-CGCTTCACGAATTTGCGT-3′
	reverse	5′-CTCGCTTCG CAGCACA-3′

### Animal experiment

The protocol has been previously described [19]. In brief, SPF female nude mice, aged 4–5 weeks, were purchased from Shanghai Shrek Experimental Animal Center (Shanghai, China). The nude mice were kept in separate cages, with three nude mice per group. A total of 2 × 10^6^ MAD-MB-231 cells stably transfected with miR-543 agomir (GenePharma, China) or NC agomir (GenePharma, China) were injected in the abdomens of nude mice. Next, the mice were maintained for 35 d, and in vivo tumor formation was recorded daily. At the end of this experiment, the mice were sacrificed, and the tumors were excised, measured, and photographed.

### Luciferase assay

The wild‐type UBE2T and the mutant form were subcloned into reporter plasmid pmirGLO (Promega, USA), then co-transfected with miR-543 mimics or NCs into breast cancer cells. Based on a published study [20], luciferase activity was measured by the Dual-Luciferase Reporter System (Promega, USA). The luminescence intensity of firefly luciferase was normalized to that of *Renilla* luciferase.

### CCK-8

Cell viability was observed from 24 h to 96 h every 24 h after transfections using a TransDetect CCK (TransGen Biotech, China). According to a previously published protocol [20], the cells used to conduct the CCK-8 assay were seeded into a 96-well plate for transfection. Absorbance at 450 nm was observed via a microplate reader (Bio-Rad, USA).

### BrdU assay

Cell proliferation was determined using a BrdU Cell Proliferation Assay Kit (CST, USA). Similar to a previously published protocol [7], the cells were seeded into a 96-well plate to conduct transfection. The BrdU assay was performed 48 h after the transfection. BrdU solution (10 μM per well) was added to incubate the transfected cells for 2 h. Following the incubation, the cells were placed in a fixing solution for 30 min at room temperature. Next, the cells were treated with anti-BrdU-antibody (1:500, Sigma-Aldrich, USA) for 1 h at room temperature. Subsequently, a FITC‐labeled goat anti-rabbit fluorescent secondary antibody was used to incubate the cells for another hour at room temperature. Finally, the absorbance values were measured at 450 nm using a fluorescence microscope (Olympus, Japan).

### Cell migration assay

The cells were seeded into a 6-well plate in a 100% confluence to conduct the wound healing assay. As previously described [20], a wound was created with a 200 μL pipette tip. Next, the cells were cultured in succession using a serum-free medium for 1 d and recorded under a microscope (Olympus, Japan). The photomicrographs were analyzed using ImageJ software. Cell migration rate (%) = (0 h scratch width – 24 h scratch width)/0 h scratch width × 100%.

### Cell invasion assay

As previously described [21], the performance was determined on 24-well Transwell chambers coated with Matrigel (BD, USA). Specifically, 1 × 10^4^ breast cancer cells mixed with 200 μL media were placed in the upper chamber. However, only media (600 μL) without cells were set in the lower chamber. When the 24 h incubation was completed, 4% paraformaldehyde was applied to immobilize the migrated cells (approximately 20 min). Then, 0.1% crystal violet was applied for staining (5 min). Cell counting was accomplished with an inverted microscope (Olympus, Japan).

### Western blotting

As previously described [22], protein extraction was conducted using RIPA buffer (Sigma‐Aldrich, USA). Equal amounts of protein samples were separated using 12% SDS‐polyacrylamide gel electrophoresis. When the protein separation was complete, polyvinylidene difluoride membranes (Invitrogen, USA) were used for the protein transfer. Next, the samples were blocked in 5% skim milk (2 h). When the block was completed, anti-UBE2T (1;1000, Cat#: ab154022, Abcam, UK), anti-ERK, anti-p-ERK, anti-RSK2, anti-p-RSK2 (1:800; Cell Signaling Technology, Danvers, MA, USA), anti-β-actin (1:1000; Abcam, Cambridge, MA), and anti‐GAPDH (1;1000, Cat#: ab8245, Abcam, UK) were used to treat the samples for overnight at 4°C. The next morning, the samples were treated with a secondary antibody (1 h). At the end, protein bands were observed with an Odyssey instrument (Li-cor, USA).

### RNA pull-down assay

Biotin-labeled miR-543 (Bio-miR-543, 5ʹ-UGGCG GAGAACUGAUAAGGGUCCUUAUCAGUUCUCCGUCCAUU-biotin-3ʹ) and negative control random RNA (Bio-NC, 5ʹ-UUCUCCGAACGUG UCACGUTTACGUGACACGUUCGGAGAATT-biotin-3ʹ) were synthesized by Sangon Biotech (Shanghai, China). As previously described [23], 50 µL Bio-miR-543 or Bio-NC was first incubated with the cell lysate (500 µL). Then, the complexes were isolated by streptavidin agarose beads (Invitrogen, USA), and qRT-PCR was used to analyze the mRNA abundance.

### Data analysis

The results are presented as the mean ± standard error of the mean of each set of three independent experiments. Student’s t-test and an analysis of variance (ANOVA) were used to determine the statistical significance of differences. *P < 0.05 was considered statistically significant.

## Results

All the above methods were conducted to explore the molecular mechanism of miR-543 and UBE2T in breast cancer progression. The results allowed us to demonstrate that miR-543 works as an anti-tumor regulator in breast cancer cells by downregulating the expression of UBE2T, whose high expression can enhance the proliferation and malignancy of breast cancer.

### miR-543 and UBE2T can be key players in breast cancer

**Figure 1.**
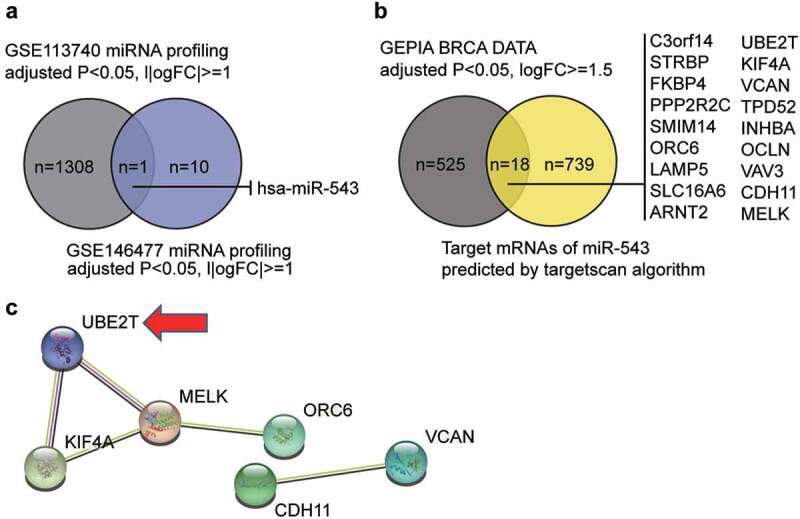
**miR-543 and UBE2T can be key players in breast cancer**. (a) The Venn diagram showing the intersection between the GSE113740 miRNA profiling and the GSE146477 miRNA profiling. FC: fold-change. (b) The Venn diagram showing the intersection between the GEPIA breast cancer data and the predicted target mRNAs of miR-543 by targetscan algorithm. FC: fold-change. (c) The PPI analysis of the mRNAs in Figure B using STRING algorithm. Line color indicates the type of interaction evidence

By intersecting the miRNA list of GSE113740 (selection criteria: adjusted P < 0.05, |logFC|≥1) and that of GSE146477 (selection criteria: adjusted P < 0.05, |log_2_FC|≥1), miR-543 was identified as the candidate miRNA that may participate in breast cancer pathogenesis (Figure 1a). miR-543 was a significant suppressor of breast cancer [6,24,25]. Nonetheless, the study of miR-543 in breast cancer is still limited. We also identified candidate downstream mRNA targets of miR-543 using the targetscan algorithm (http://targetscan.org/vert_72) and interrogating GEPIA breast cancer data (adjusted P < 0.05, log_2_FC≥1.5) (Figure 1b). Eighteen candidates were identified and uploaded to the STRING database for PPI network analysis (Figure 1c). The PPI network showed that six of the 18 candidate genes were closely associated. We noticed that UBE2T was once reported to be a proliferation promoter in breast cancer [13,14], but the effects of UBE2T on other cell phenotypes have not been reported, nor has whether UBE2T can be regulated by miRNAs. Thus, affected cell phenotypes have not been reported.

### miR-543 inhibited cell viability and proliferation in breast cancer

**Figure 2.**
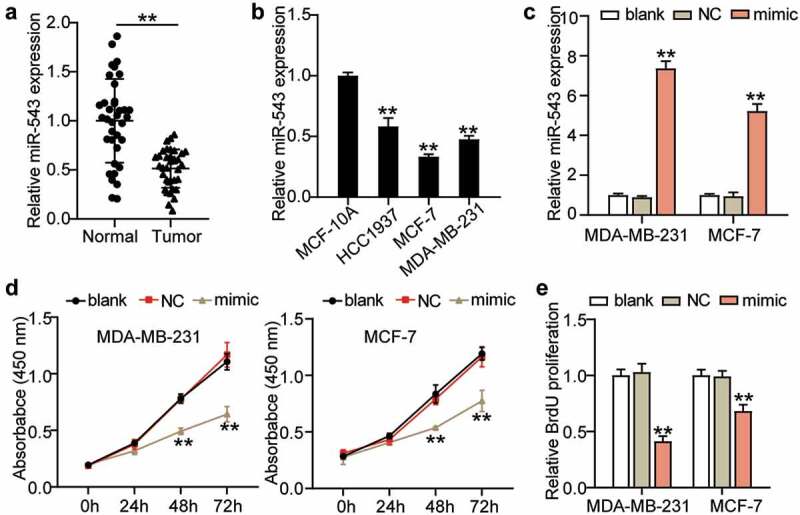
**miR-543 inhibited cell viability and proliferation in breast cancer**. (a) Expression level of miR-543 in breast cancer tissues and normal tissues by qRT-PCR. **P < 0.001 versus normal tissues, n = 36. (b) miR-543 expression was also measured in Human normal mammary epithelial cells (MCF-10A) and breast cancer cell lines (HCC1937, MCF-7, MDA-MB-231). **P < 0.001 versus MCF-10A cells. (c) miR-543 mimics transfection efficiency was detected by qRT-PCR. (d) Cell viability was detected by CCK-8 assay. (e) Cell proliferation was detected by BrdU assay. (c-e) **P < 0.001 versus blank control group. Blank: blank control. NC: negative control. mimic: miR-543 mimic

To detect the expression profile and function of miR-543 in breast cancer, first, the expression level of miR-543 was determined in breast cancer tissues and normal tissues by qRT-PCR. This revealed a 50% decreased level of miR-543 in breast cancer tissues, indicating an inhibitory role of miR-543 in breast cancer (Figure 2a). Furthermore, miR-543 expression was also measured in MCF-10A and breast cancer cell lines (HCC1937, MCF-7, MDA-MB-231). As expected, a significant downregulation of miR-543 was detected in breast cancer cell lines, and MCF-7 and MDA-MB-231 cells exhibited the most significant downregulation of miR-543 (more than 50%) in the breast cancer cell lines (Figure 2b). For this reason, these two types of cells were chosen for the following experiments. miR-543 mimics were synthesized to overexpress miR-543. The transfection efficiency is depicted in Figure 2c. It was shown that miR-543 mimics successfully upregulated miR-543 expression by more than 5-fold (Figure 2c). Subsequently, the action of miR-543 in the MCF-7 and MDA-MB-231 cells was explored. Cell viability was dramatically restrained by miR-543 enrichment (Figure 2d). Furthermore, proliferation was dramatically restrained by over 50% or approximately 25% in these two cell lines in the miR-543 mimic group (Figure 2e). Collectively, miR-543 had a negative effect on cell viability and proliferation in breast cancer.

### miR-543 had a negative effect on cell migration and invasion in breast cancer

**Figure 3.**
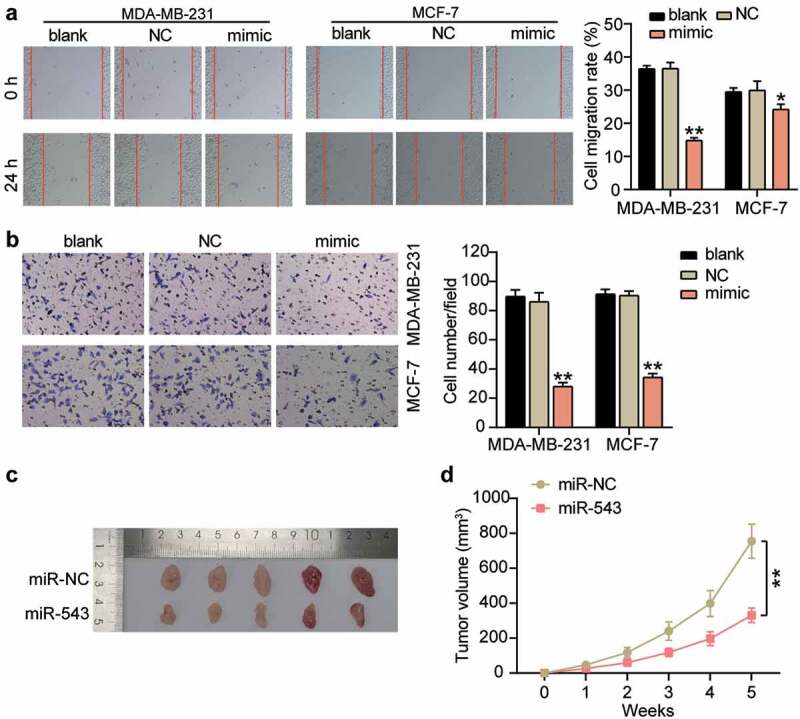
**miR-543 had a negative effect on cell migration and invasion in breast cancer**. (a) Cell migration was detected by wound healing assay. (b) Cell invasion was detected by transwell invasion assay. (a-b) *P < 0.05, **P < 0.001 versus blank control group. Blank: blank control. NC: negative control. mimic: miR-543 mimic. (c) Subcutaneous tumors in nude mice derived with MDA-MB-231 cells transfected with miR-543 agomir and NC agomir. (d) tumor volumes of the subcutaneous tumors. **P < 0.001. NC: negative control

Cell migration and invasion are vital processes in cancer cell growth and development. Therefore, we investigated the action of miR-543 in these two cell processes in breast cancer cells to further illustrate its effects on breast cancer progression. Our results showed that when miR-543 was overexpressed, cell migration ability was repressed by approximately 50% and 15% in MDA-MB-231 and MCF-7 (Figure 3a), compared with the blank control group. Cell invasion was suppressed by approximately 65% in MDA-MB-231 cells and approximately 60% in MCF-7 cells (Figure 3b), compared to the blank control group. Additionally, animal experiments showed that miR-543 inhibited tumor formation in vivo (Figures 3C, 3D). Thus, miR-543 exhibited a negative effect on these two cell processes in breast cancer.

### miR-543 targeted UBE2T and reduced UBE2T expression in breast cancer cells

**Figure 4.**
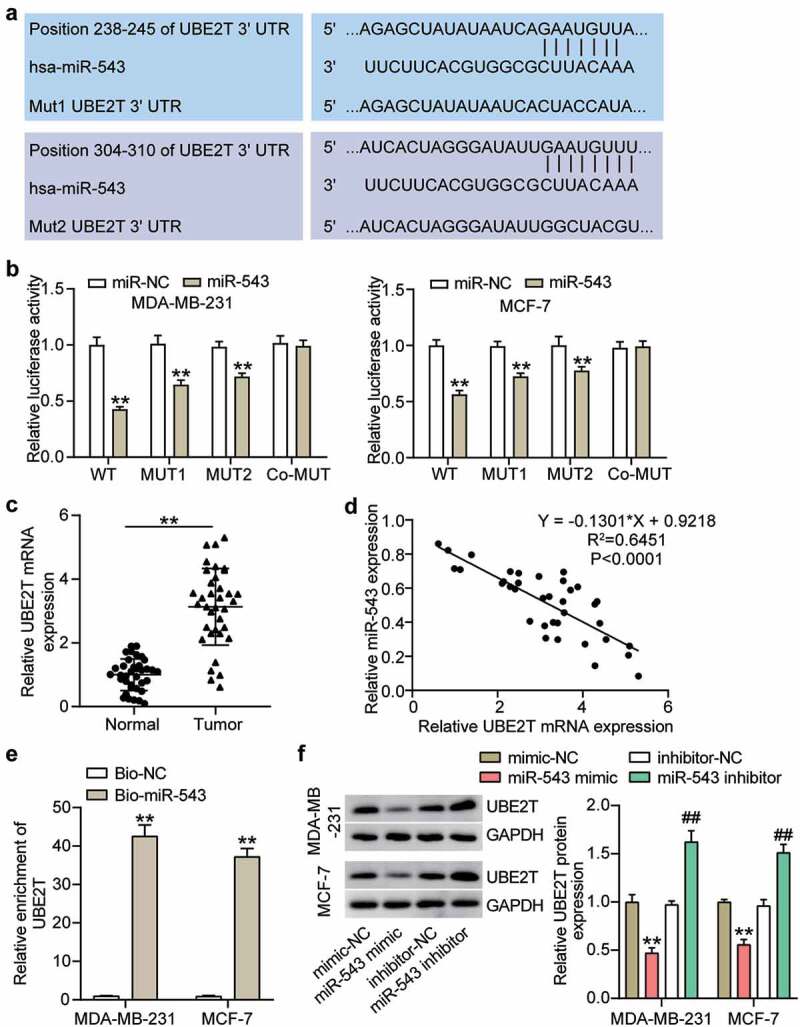
**miR-543 targeted UBE2T in breast cancer cells**. (a) The binding sites between miR-543 and UBE2T were predicted by TargetScan. (b) Dual-luciferase reporter assay was conducted to confirm the relationship between miR-543 and UBE2T. **P < 0.001 versus miR-NC group. NC: negative control. WT: wild type, MUT: mutation. (c) Expression level of UBE2T in breast cancer tissues and normal tissues by qRT-PCR. **P < 0.001. n = 36. (d) Pearson’s correlation analysis was used to determine the relationship between expression of miR-543 and UBE2T in breast cancer tissues. (e) RNA pull-down assay was carried out to quantify the UEB2T enrichment in MDA-MB-231 and MCF-7 transfected with bio-miR-543 or bio-miR-NC. Bio-miR-543: Biotin-labeled miR-543. Bio-NC: Biotin-labeled negative control. **P < 0.001 versus bio-NC group. (f) The expression level of UBE2T in MDA-MB-231 and MCF-7 transfected with miR-543 mimic or inhibitor was detected by Western blot, **P < 0.001 versus mimic-NC group. **P < 0.001 versus inhibitor-NC group

It is well established that miRNAs exert function typically by deregulating target gene expression. In this regard, we searched the potential target genes of miR-543 and found that UBE2T was a potential target gene of miR-543 (Figure 4a). There were two matching sites between miR-543 and UBE2T (Figure 4a). An obvious suppression of luciferase activity was observed in the UBE2T wild type group, with a relatively less of a significant change in the mutation of only one binding site than the wild type group (Figure 4b). There was no observable reduction of luciferase activity in the co-mutant group (Figure 4b). This verified that miR-543 could target UBE2T in breast cancer cells. Additionally, UBE2T exhibited the opposite level to miR-543 in breast cancer and healthy tissues, indicating that UBE2T expression in breast cancer tissues was 2-fold higher than that of normal tissues (Figure 4c). Furthermore, Pearson’s correlation analysis showed that UBE2T expression was negatively related to miR-543 expression in breast cancer tissues (Figure 4d). Compared with the Bio-NC, the enrichment of UBE2T was increased in the Bio-miR-543 group, which suggested that miR-543 directly targeted UBE2T in breast cancer cells (Figure 4e). Additionally, Western blot was performed to demonstrate that the UBE2T expression level was significantly decreased in MCF-7 and MDA-MB-231 cells transfected with the miR-543 mimic, whereas increased expression was observed in MCF-7 and MDA-MB-231 cells transfected with the miR-543 inhibitor compared with the corresponding negative control (figure 4f). Collectively, these results verified that miR-543 directly targeted UBE2T and suppressed UBE2T expression in breast cancer cells.

### miR-543 repressed UBE2T to inhibit cell viability and proliferation in breast cancer cells

**Figure 5.**
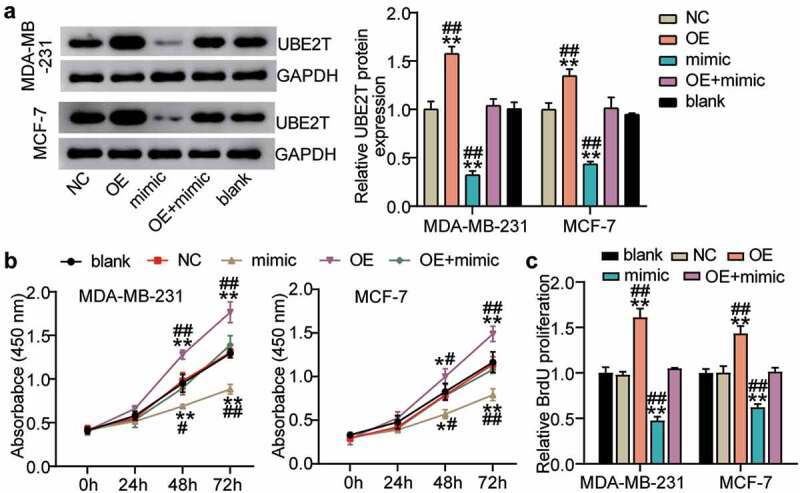
**miR-543 repressed UBE2T to inhibit cell viability and proliferation in breast cancer cells**. (a) The transfection efficiency of UBE2T overexpression plasmid was detected by Western blotting. (b) Cell viability was detected by CCK-8 assay. (c) Cell proliferation was detected by BrdU assay. **P < 0.001 versus blank group. #P < 0.05, **P < 0.001 versus OE+mimic group. Blank: blank control. NC: negative control. OE: UBE2T overexpression vector. Mimic: miR-543 mimic

Because miR-543 was able to target UBE2T in breast cancer cells, confirming whether miR-543 could exert a function interacting with UBE2T was conducted. The results suggested that in the MDA-MB-231 cell line compared to the blank control, UBE2T protein expression was enriched by more than 50% in the UBE2T overexpression group, whereas miR-543 mimics suppressed UBE2T protein expression by approximately 60%. Additionally, UBE2T overexpression plus miR-543 mimics compared with the blank group caused no significant change in UBE2T protein expression level (Figure 5a). There was a similar trend for MCF-7 cells as that in MDA-MB-231 cells (Figure 5a). Cell viability was promoted by UBE2T overexpression, whereas miR-543 mimics reversed this effect (Figure 5b). Cell proliferation was facilitated by over 50% in the UBE2T overexpression group, whereas the facilitation was compromised by miR-543 mimics (Figure 5c). Taken together, miR-543 repressed UBE2T to inhibit cell viability and proliferation in breast cancer cells.

**miR-543 inhibited cell migration and invasion in breast cancer cells via suppressing UBE2T through the ERK/MAPK pathway**

**Figure 6.**
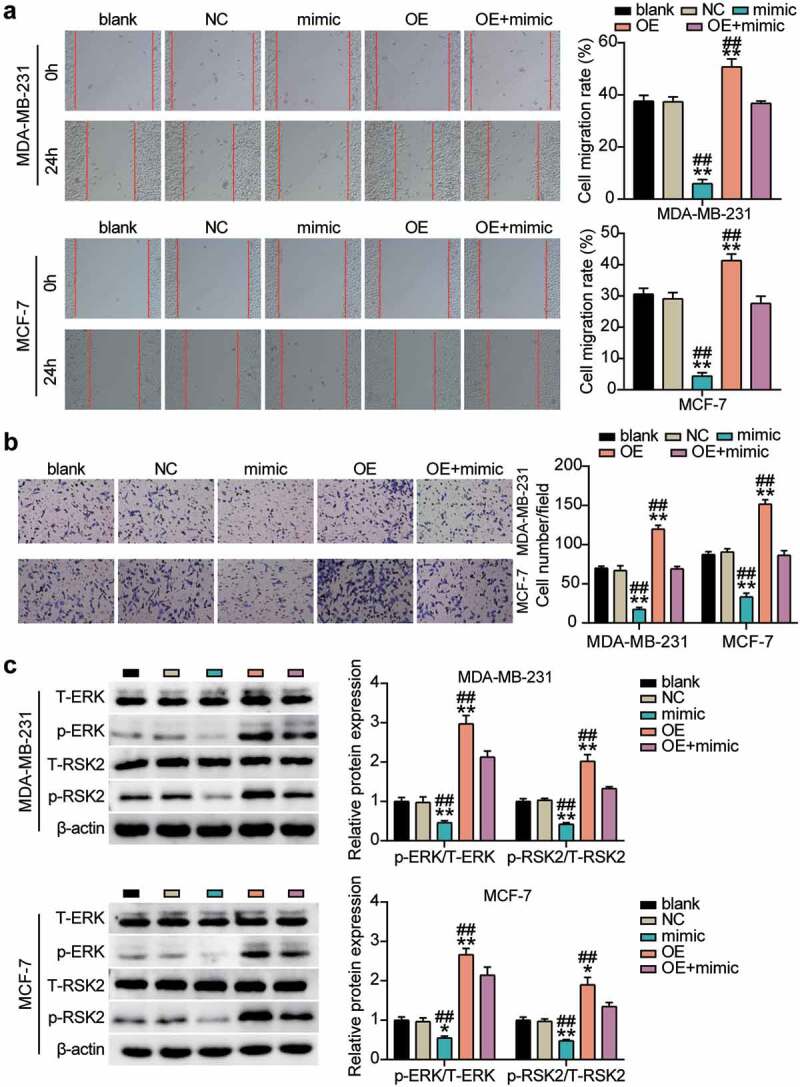
**miR-543 repressed UBE2T to inhibit cell migration and invasion in breast cancer cells**. (a) Cell migration was detected by wound healing assay. (b) Cell invasion was detected by transwell invasion assay. **P < 0.001 versus blank group. **P < 0.001 versus OE+mimic group. Blank: blank control. NC: negative control. OE: UBE2T overexpression vector. Mimic: miR-543 mimic. (c) Western blot experiment was performed to detect the activity of ERK/MAPK pathway. **P < 0.001 vs negative control

Similar to the confirmation of the action of miR-543 in cell migration and invasion in breast cancer cells, we also explored the role of UBE2T through rescued experiments. The experimental results showed that compared with NCs, cell migration was promoted by approximately 25% to 30% in the UBE2T overexpression group, whereas miR-543 mimics could reverse this effect (Figure 6a). In comparison with NCs, cell invasion was facilitated by approximately 50% in the UBE2T overexpression group, whereas the facilitation could be rescued by miR-543 mimics (Figure 6b). Western blot data indicated that the levels of phosphorylated proteins (ERK and RSK) were upregulated in the UBE2T overexpression cells, whereas the significant effects were restored by co-transfecting with the miR-543 mimic (Figure 6c). Overall, these data indicated that the tumor-inhibitory effect of miR-543 was executed by downregulating UBE2T through the ERK/MAPK pathway.

## Discussion

Our results implied that miR-543 had reduced expression in breast cancer tissues, whereas its targeted gene UBE2T exhibited higher expression in breast cancer tissues. Furthermore, miR-543 inhibited breast cancer cell viability and cell proliferation via repressing UBE2T expression. Additionally, the miR-543/UBE2T axis also participated in regulating breast cancer cell migration and invasion, which could be suppressed by miR-543 but promoted by UBE2T. Overall, miR-543 alleviated breast cancer progression and malignancy through the regulation of UBE2T.

Considerable studies showed that miRNAs act as important regulators in carcinogenesis [26,27]. Therefore, exploration of the expression and effects of miRNAs as diagnostic and prognostic markers is meaningful [28,29]. Here, we focused on the part that miR-543 takes in breast cancer progression, attempting to provide additional new information regarding biotarget therapy for breast cancer. Previously, lower miR-543 expression was reported in endometrial cancer [30] and glioma [31], which is in accordance with the present observation that miR-543 displayed a reduced level in breast cancer progression. In the present study, functionally, miR-543 acted as an anti-tumor miRNA in breast cancer and participated in regulating cell viability, proliferation, migration, and invasion. Furthermore, miR-543 expression was previously revealed to be significantly decreased in breast cancer tissues, which showed the metastasis-suppressive role of miR-543 in breast cancer. For example, Ji and colleagues demonstrated that miR-543 inhibits the EMT-like phenotype and TGF-β-induced breast cancer metastasis both in vitro and in vivo by targeting ZNF218 [24]. Wang and et al. illustrated that miR-543 inhibited the malignant behavior of triple-negative breast cancer by directly targeting ACTL6A and suppressing ACTL6A expression [19]. Additionally, Li et al. found that miR-543 impaired cell proliferation, migration, and invasion in breast cancer via repressed VCAN [7], which is primarily consistent with the viability, proliferation, migration, and invasion-suppressive role of miR-543 in breast cancer in this study. However, Li and colleagues mainly focused on the regulation mechanism of miR-543 and CAN, which participates in the extracellular matrix formation. However, we demonstrated that miR-543 inhibited the malignant development of breast cancer by targeting UBE2T through the ERK/MARK signaling pathway.

It has been highlighted that UBE2T is an essential factor in protein ubiquitination, mediating multiple biological processes, such as inflammation, immune response, cell growth, and carcinogenesis. The involvement of proteins in ubiquitination and degradation influences drug susceptibility of cancer cells. Based on this, it is possible to develop therapeutic drugs to investigate key factors essential in protein ubiquitination [32]. Therefore, a high level of UBE2T has been depicted in different kinds of cancer, including lung cancer [33], hepatocellular carcinoma [15], and renal cell carcinoma [34], suggesting the tumor-promoting role of UBE2T, which has the potential to act as a promising drug target in cancer therapy. Therefore, it is reasonable to regard UBE2T as a promising drug target for patients with breast cancer. Prior studies have published that UBE2T was overexpressed and linked with undesirable outcome in breast cancer patients [14]. Ueki and et al. found that UBE2T enhanced the development of breast cancer cells by downregulating BRCA1 expression [13]. However, its detailed effect on breast cancer progression, such as cell proliferation and migration, must be verified. In this study, our experimental verification confirmed the high UBE2T expression in breast cancer and demonstrated its significant function in the development and progression of breast cancer, which was regulated and restored with the introduction of miR-543. Furthermore, we confirmed that the promotion effects of UBE2T on breast cancer malignancy by inhibiting ERK/MAPK activity. In accordance with the present results, previous studies have demonstrated that UBE2T was directly targeted and downregulated by miR-543, which attenuated the proliferation and progression of hepatocellular carcinoma [15]. Overall, our study first demonstrated that the axis of miR-543/UBE2T in the development of breast cancer by inhibiting the ERK/MAPK pathway, which indicated that miR-543, UBE2T, and their regulation of the ERK/MAPK pathway, which could have prognostic and diagnostic values for patients with breast cancer.

However, despite the mechanical demonstration of the miR-543/UBE2T axis in the breast cancer process in this study, the effect of miR-543 and UBE2T is lacking in vitro verification. Additionally, the upstream regulator of miR-543/UBE2T should be explored in future studies.

## Conclusion

This research depicted that miR-543 targeted and repressed the expression of UBE2T to inhibit the ERK/MAPK pathway, which eventually inhibited the malignant behavior of breast cancer cells. Therefore, miR-543 and UBE2T could be promising prognostic targets for breast cancer. This study provided insights into understanding the regulatory mechanism underlying miR-543 and UBE2T in the development of breast cancer.

## Data Availability

The datasets used and analyzed during the current study are available from the corresponding author on reasonable request.

## Abbreviations

ERK, extracellular signal-regulated kinase; MAPK, mitogen-activated protein kinase.

## References

[1] Woolston C. Breast cancer. Nature. 2015;527(7578):S101. 26580154 10.1038/527S101a

[2] Pearce L. Breast cancer. Nursing standard (Royal College of Nursing (Great Britain): 1987). 2016;30(51):15. 10.7748/ns.30.51.15.s16 27533387

[3] Fahad Ullah M. Breast cancer: Current perspectives on the disease status. Adv Exp Med Biol. 2019;1152:51–64. 31456179 10.1007/978-3-030-20301-6_4

[4] Wörmann B. Breast cancer: basics, screening, diagnostics and treatment. Medizinische Monatsschrift fur Pharmazeuten. 2017;40(2):55–64. 29952495

[5] Romero-Cordoba S, Rodriguez-Cuevas S, Rebollar-Vega R, et al. Identification and pathway analysis of microRNAs with no previous involvement in breast cancer. PLoS One. 2012;7(3):e31904. 22438871 10.1371/journal.pone.0031904 PMC3306365

[6] Chen P, Xu W, Luo Y, et al. MicroRNA 543 suppresses breast cancer cell proliferation, blocks cell cycle and induces cell apoptosis via direct targeting of ERK/MAPK. Onco Targets Ther. 2017;10:1423–1431. 28331335 10.2147/OTT.S118366 PMC5348068

[7] Li R, Hou S, Zou M, et al. miR-543 impairs cell proliferation, migration, and invasion in breast cancer by suppressing VCAN. Biochem Biophys Res Commun. 2021;570:191–198. 34293593 10.1016/j.bbrc.2021.07.005

[8] Alpi AF, Chaugule V, Walden H. Mechanism and disease association of E2-conjugating enzymes: lessons from UBE2T and UBE2L3. Biochem J. 2016;473(20):3401–3419. 27729585 10.1042/BCJ20160028 PMC5095918

[9] Rickman KA, Lach FP, Abhyankar A, et al. Deficiency of UBE2T, the E2 ubiquitin ligase necessary for FANCD2 and FANCI ubiquitination, causes FA-T subtype of fanconi anemia. Cell Rep. 2015;12(1):35–41. 26119737 10.1016/j.celrep.2015.06.014 PMC4497947

[10] Lim KH, Song MH, Baek KH. Decision for cell fate: deubiquitinating enzymes in cell cycle checkpoint. Cell Mol Life Sci. 2016;73(7):1439–1455. 26762302 10.1007/s00018-015-2129-2 PMC11108577

[11] Machida YJ, Machida Y, Chen Y, et al. UBE2T is the E2 in the Fanconi anemia pathway and undergoes negative autoregulation. Mol Cell. 2006;23(4):589–596. 16916645 10.1016/j.molcel.2006.06.024

[12] Fuentes-Antrás J, Alcaraz-Sanabria AL, Morafraile EC, et al. Mapping of genomic vulnerabilities in the post-translational ubiquitination, SUMOylation and neddylation machinery in breast cancer. Cancers (Basel). 2021;13:4. 10.3390/cancers13040833 PMC7922122 33671201

[13] Ueki T, Park JH, Nishidate T, et al. Ubiquitination and downregulation of BRCA1 by ubiquitin-conjugating enzyme E2T overexpression in human breast cancer cells. Cancer Res. 2009;69(22):8752–8760. 19887602 10.1158/0008-5472.CAN-09-1809

[14] Perez-Peña J, Corrales-Sánchez V, Amir E, et al. Ubiquitin-conjugating enzyme E2T (UBE2T) and denticleless protein homolog (DTL) are linked to poor outcome in breast and lung cancers. Sci Rep. 2017;7(1):17530. 29235520 10.1038/s41598-017-17836-7 PMC5727519

[15] Liu LP, Yang M, Peng QZ, et al. UBE2T promotes hepatocellular carcinoma cell growth via ubiquitination of p53. Biochem Biophys Res Commun. 2017;493(1):20–27. 28935368 10.1016/j.bbrc.2017.09.091

[16] Guo YJ, Pan WW, Liu SB, et al. ERK/MAPK signalling pathway and tumorigenesis. Exp Ther Med. 2020;19(3):1997–2007. 32104259 10.3892/etm.2020.8454 PMC7027163

[17] Yu Y, Luo W, Yang ZJ, et al. miR-190 suppresses breast cancer metastasis by regulation of TGF-β-induced epithelial-mesenchymal transition. Mol Cancer. 2018;17(1):70. 29510731 10.1186/s12943-018-0818-9 PMC5838994

[18] Livak KJ, Schmittgen TD. Analysis of relative gene expression data using real-time quantitative PCR and the 2(-delta delta C(T)) method. Methods. 2001;25(4):402–408. 11846609 10.1006/meth.2001.1262

[19] Wang YL, Liang RH, Wang CY, et al. MicroRNA-543 inhibits the proliferation, migration, invasion, and epithelial-mesenchymal transition of triple-negative breast cancer cells via down-regulation of ACTL6A gene. Clin Transl Oncol. 2021. doi:10.1007/s12094-021-02672-z 34181232

[20] Xu Y, Wang J, Wang J. Long noncoding RNA XIST promotes proliferation and invasion by targeting miR-141 in papillary thyroid carcinoma. Onco Targets Ther. 2018;11:5035–5043. 30174441 10.2147/OTT.S170439 PMC6110635

[21] Liu A, Liu L, Lu H. LncRNA XIST facilitates proliferation and epithelial-mesenchymal transition of colorectal cancer cells through targeting miR-486-5p and promoting neuropilin-2. J Cell Physiol. 2019;234(8):13747–13761. 30656681 10.1002/jcp.28054 PMC13483007

[22] Wang Y, Liu Z, Ren X, et al. Hsa-miR-330-5p Aggravates Thyroid Carcinoma via Targeting FOXE1. J Oncol. 2021;2021:1070365. 34306074 10.1155/2021/1070365 PMC8272668

[23] Yamamoto K, Ito S, Hanafusa H, et al. Uncovering direct targets of MiR-19a involved in lung cancer progression. PLoS One. 2015;10(9):e0137887. 26367773 10.1371/journal.pone.0137887 PMC4569347

[24] Ji W, Mu Q, Liu XY, et al. ZNF281-miR-543 feedback loop regulates transforming growth factor-β-induced breast cancer metastasis. Mol Ther Nucleic Acids. 2020;21:98–107. 32512343 10.1016/j.omtn.2020.05.020 PMC7281305

[25] Wang H, Huang Y, Yang Y. LncRNA PVT1 regulates TRPS1 expression in breast cancer by sponging miR-543. Cancer Manag Res. 2020;12:7993–8004. 32982402 10.2147/CMAR.S263383 PMC7493016

[26] Khan AQ, Ahmed EI, Elareer NR, et al. Role of miRNA-regulated cancer stem cells in the pathogenesis of human malignancies. Cells. 2019;8:8. 10.3390/cells8080840 PMC6721829 31530793

[27] Kabekkodu SP, Shukla V, Varghese VK, et al. Clustered miRNAs and their role in biological functions and diseases. Biol Rev Camb Philos Soc. 2018;93(4):1955–1986. 29797774 10.1111/brv.12428

[28] Mollaei H, Safaralizadeh R, Rostami Z. MicroRNA replacement therapy in cancer. J Cell Physiol. 2019;234(8):12369–12384. 30605237 10.1002/jcp.28058 PMC13484118

[29] Babaei K, Shams S, Keymoradzadeh A, et al. An insight of microRNAs performance in carcinogenesis and tumorigenesis; an overview of cancer therapy. Life Sci. 2020;240:117077. 31751586 10.1016/j.lfs.2019.117077

[30] Bing L, Hong C, Li-Xin S, et al. MicroRNA-543 suppresses endometrial cancer oncogenicity via targeting FAK and TWIST1 expression. Arch Gynecol Obstet. 2014;290(3):533–541. 24699721 10.1007/s00404-014-3219-3

[31] Ji T, Zhang X, Li W. MicroRNA‑543 inhibits proliferation, invasion and induces apoptosis of glioblastoma cells by directly targeting ADAM9. Mol Med Rep. 2017;16(5):6419–6427. 28849046 10.3892/mmr.2017.7332

[32] Pagliarini R, Shao W, Sellers WR. Oncogene addiction: pathways of therapeutic response, resistance, and road maps toward a cure. EMBO Rep. 2015;16(3):280–296. 25680965 10.15252/embr.201439949 PMC4364868

[33] Hao J, Xu A, Xie X, et al. Elevated expression of UBE2T in lung cancer tumors and cell lines. Tumour Biol. 2008;29(3):195–203. 18667844 10.1159/000148187

[34] Hao P, Kang B, Li Y, et al. UBE2T promotes proliferation and regulates PI3K/Akt signaling in renal cell carcinoma. Mol Med Rep. 2019;20(2):1212–1220. 31173226 10.3892/mmr.2019.10322 PMC6625406

